# Evaluation of Cone Beam Computed Tomography (CBCT) System: Comparison with Intraoral Periapical Radiography in Proximal Caries Detection

**DOI:** 10.5681/joddd.2012.001

**Published:** 2012-03-13

**Authors:** Solmaz Valizadeh, Mohammad Amin Tavakkoli, Homaoun Karimi Vasigh, Zeynab Azizi, Tara Zarrabian

**Affiliations:** ^1^Assistant Professor, Department of Oral and Maxillofacial Radiology, Faculty of Dentistry, Shahid Beheshti University of Medical Sciences, Tehran, Iran; ^2^Professor, Department of Oral and Maxillofacial Radiology, Faculty of Dentistry, Shahid Beheshti University of Medical Sciences, Tehran, Iran; ^3^Oral and Maxillofacial Radiologist, Private Practice, Tehran, Iran; ^4^Post-graduate Student, Department of Oral and Maxillofacial Radiology, Faculty of Dentistry, Shahid Beheshti University of Medical Sciences, Tehran, Iran; ^5^Post-graduate Student, Department of Pediatric Dentistry, Dental Branch, Islamic Azad University of Tehran, Tehran, Iran

**Keywords:** Cone Beam CT, periapical imaging, proximal caries

## Abstract

**Background and aims:**

With the introduction of Cone Beam Computed Tomography (CBCT) in dentistry, a question has been raised whether the technique significantly increases the diagnostic accuracy in comparison with other techniques or not. Therefore, the present study was undertaken to assess the accuracy of CBCT modality in detecting proximal carious lesions as compared to conventional periapical radiographs.

**Materials and methods:**

This diagnostic study was carried out on 84 human extracted molars and premolars. The teeth were mounted and divided in 28 blocks of 3 teeth. Periapical and CBCT images of teeth were obtained. Five observers scored the images for the detection of proximal carious lesions using a 2-point scale (caries, present; caries, absent). The gold standard was determined by histopathologic sections. Sensitivity, specificity, PPV, NPV and receiver operating charac-teristics (ROC) curves were traced for observers in both systems. The results were analyzed by paired t-test.

**Results:**

The area under the ROC curve, sensitivity, specificity, accuracy, positive and negative predictive values of CBCT images were 0.568, 0.835, 0.637, 0.714, 0.598 and 0.856, respectively. These parameters were 0.432, 0.837, 0.722, 0.77, 0.687 and 0.858 for the periapical conventional technique, respectively.

**Conclusion:**

The CBCT images did not enhance detection of proximal caries in comparison with periapical images.

## Introduction

Different modalities have been introduced to detect and outline carious lesions and evaluate their progression and efficacy of the treatment. Difficulties in radiographic detection of carious lesions is a chief concern when considering a treatment plan. ^1^ Considering the decrease in the incidence, size and progression process, it is very important to provide more accurate methods. ^1^ Caries is usually detected by bitewing radiography or visual inspection, but non-cavitated lesions may not initially be detected precisely, leading to large lesions.^2 , 3^ These lesions enlarge slowly and are not visible on radiographs until they extend beyond half the enamel thickness. Nevertheless, approximately 40% of demineralization should occur for caries to be detected on images.^4^

In recent years digital systems are widely applied because of the manipulation ability in contrast, brightness and magnification. Several studies have showed that diagnostic accuracy of this technique is equal or even slightly more than conventional methods.^5 - 8^ But misdiagnosis also occurs in digital radiographs in slightly demineralized and small lesions as the result of low contrast resolution. In general, digital radiography has no superiority over the conventional method in proximal caries detection.^7 , 9 - 11^

Cone beam computed tomography (CBCT) is a new method which uses the reciprocal rotation of a two-dimensional receptor and a cone-shaped x-ray beam to gain volume data.^12^ With a slight dose increase in this method, a large amount of high quality data is collected and in most cases this increase in dose is acceptable to obtain accurate information. CBCT doses are different according to the manufacturer, exposure parameters and observer ability.^13^ CBCT application currently extends to almost all dentistry fields, including implantology, craniofacial surgery, endodontics, orthodontics, periodontology and so on. However, there are concerns about the excess dose if there would not be much advantage in the diagnosis and treatment. The recent technology might help detect proximal caries but its priority should be taken into account in addition to its higher dose range; to date, only a small amount of research has been devoted to detection of caries using this system; according to literature, it is still a matter of controversy whether CBCT is superior to conventional modalities in the diagnosis of dental caries at this stage. Since only in one of the studies our type of CBCT system has been used the present study was undertaken to determine the capacity of this type of CBCT system for detection of caries.

## Materials and Methods

In this diagnostic in vitro study, eighty-four extracted molar and premolar teeth, with no gross cavitations or restorations but with proximal caries (white spot or small cavitation not in gross form) in at least one of the surfaces, were selected. The teeth (including two premolars and one molar) were mounted in plaster and divided into 28 blocks of 3 teeth. Twenty-eight periapical images and 7 CBCT images were obtained. Each four block was placed on the chin rest of the CBCT system, so there was one scan for each four block. The periapical radiographs of teeth were taken in a fixed standard condition by parallel technique, with 65 kVp and 10 mA, 32 cm OSD (object-to-source distance), 2 cm OID (object-to-image receptor distance), using E-speed films (AGFA, Heraeus Kulzer GmbH; Hanau, Germany). A 12-cm-thick acrylic plate was placed between the source and the teeth to simulate the clinical situation. The radiographs were processed automatically (Gendex; Clarimat 300, London, England). CBCT images were taken by NewTom 3 system (NewTom 3G V.G.QR, Inc, Verona, Italy) with 110 kVp, 1.98 mA, 3.6 s, 9 inch FOV and 0.3 mm resolution. All the images (conventional and CBCT images) were evaluated by five observers (four radiologists and a resident) in three planes of axial, coronal and sagittal. Conventional images were evaluated in the same viewbox and the CBCT images were observed in the same monitor and magnified and manipulated for contrast resolution, brightness and γ value by the observers. They assessed the images with no time limitation and randomly, one session for conventional and one session for CBCT images. A 2-point scale criterion (caries, present; caries, absent) was used to record the results.

The teeth were sectioned by diamond disks in mesiodistal direction, and 0.1-μm-thick slices were obtained for histopathologic evaluation as the gold standard.

## Results

The intra-class correlation coefficient (ICC) showed a high inter-observer agreement for both periapical radiography and CBCT images. The coefficients for CBCT and conventional radiography were 0.9259 and 0.9243, respectively. By considering the value of 1 as the complete agreement, the inter-observer agreement was excellent in both techniques.

In CBCT, the mean area under the ROC curve (AZ value) for caries detection was 0.568 for all five observers and the sensitivity and specificity were 0.835 and 0.637, respectively (Table 1). The ROC value was estimated to be 0.432, and the sensitivity and specificity were 0.837 and 0.722, respectively for the conventional radiographs (Table 2). According to the results of paired t-test, the ROC value was significantly higher in CBCT rather than the conventional method (0.432±0.029) (P<0.007). However, diagnostic sensitivities of these two methods were not significantly different (0.835±0.036 and 0.837±0.01, P=0.91); the NPV (negative predictive value) values (0.856±0.032 and 0.858±0.009, P=0.88) were not significantly different either. With paired t-test, significant superiority in specificity was obtained for conventional radiography in comparison to the CBCT (0.722±0.022 vs. 0.637±0.004, P<0.001). PPV (positive predictive value) was 0.687± and 0.598±0.004 for conventional and CBCT techniques, exhibiting a significant difference between these two methods (P<0.001). In addition, diagnostic accuracy of conventional radiography was superior to the CBCT system (0.770±0.015 vs. 0.714±0.014).

**Table 1:** Observers' results in CBCT images for area under the ROC curve, sensitivity, specificity, PPV, NPV and TP+RN

Observer	Area under the ROC curve	Sensitivity	Specificity	PPV	NPV	TP+TN
1	0.542	0.847	0.639	0.6	0.868	0.720
2	0.536	0.796	0.639	0.6	0.821	0.702
3	0.604	0.867	0.639	0.6	0.885	0.726
4	0.566	0.79	0.630	0.591	0.821	0.697
5	0.592	0.867	0.639	0.6	0.885	0.726
mean	0.568	0.835	0.637	0.598	0.856	0.714

**Table 2:** Observers results in conventional radiography images for area under the ROC curve, sensitivity, specificity, PPV, NPV and TP+RN

Observer	Area under the ROC curve	Sensitivity	Specificity	PPV	NPV	TP+TN
1	0.458	0.829	0.708	0.627	0.852	0.759
2	0.464	0.848	0.697	0.661	0.868	0.759
3	0.396	0.848	0.754	0.722	0.868	0.794
4	0.434	0.829	0.730	0.696	0.852	0.773
5	0.408	0.829	0.719	0.684	0.852	0.766
mean	0.432	0.837	0.722	0.687	0.858	0.770

**Figure 1:**
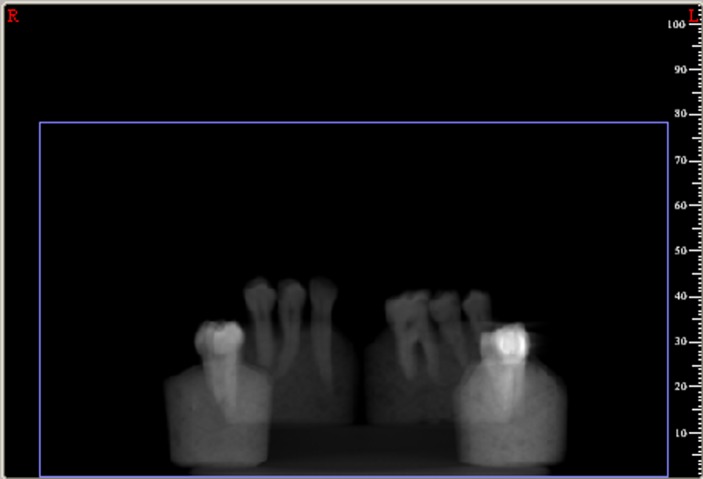
CBCT images of dental blocks.

Therefore, the periapical technique was superior to CBCT in specificity (P<0.001), positive predictive value (P<0.001) and accuracy (P<0.002) while the area under the ROC curve was higher for CBCT than the conventional technique (P<0.007). The two techniques did not show significant differences in relation to sensitivity and negative predictive value.

## Discussion

Cone beam CT is widely used in several fields of dentistry and different systems have been introduced with this technique, but more evidence is necessary to prove its superiority to conventional radiography in all the fields. Therefore, in this study diagnostic accuracy of this modality was compared with conventional technique in the detection of proximal caries. Sensitivity, specificity, accuracy, PPV, NPV and assessment of the area under ROC curve showed that both techniques had optimal ability to detect proximal caries. This CBCT system shows no higher diagnostic capacity compared to the conventional method. Some previous studies with similar subjects have yielded results similar to those of the present study. Kalathingal et al^14^ and Tsuchida et al^15^ showed no advantages for CBCT images in the detection of proximal caries compared to the conventional method. There were no significant differences in the area under the ROC curve (AZ value) between CBCT and conventional radiography in the detection of proximal caries. The ROC curve is a fundamental tool for diagnostic test evaluation. It allows a complete sensitivity/specificity report. Lack of further signs of caries being reported in CBCT images indicated similar diagnostic accuracy in these two techniques. This similarity can explained by lack of sufficient observers experience in judjing CBCT images or high qualification of them in conventional radiographs interpretation. Although some observers reported an AZ value of 0.94, the maximum value for the area under ROC curve in these similar studies was reported to be 0.604, which can be attributed to a lack of further signs of caries reported in CBCT images compared to the conventional technique. Haiter-Neto et al^16^ reported that the diagnostic accuracy of CBCT technique is the same as that of the conventional method and the reasons were reported to be the low contrast resolution, more noise, limited spatial resolution and artifacts resulting from high density structures. These factors were the same in the present study. Thin-layer detector is one of the reasons for low contrast resolution in CBCT images and few detected photons and numerous scattered radiations result in noise. In this technique the number of the photons is proportional to the tube intensity and higher intensities can lead to higher diagnostic accuracy. In this study the tube intensity was 1.98 mA with 3.6 seconds of exposure time; however, in a study by Tsuchida it was 4 mA and 18 seconds. In CBCT, generation of scattered radiation between the patient and the receptor is inevitable, although this has not been mentioned anywhere in documents. Spatial resolution, which was 0.3 mm in this study, was 0.25 mm in 3D Accutomo CBCT systems used in different studies.^17^

On the other hand some studies have shown higher diagnostic accuracy in the detection of proximal caries. Akdeniz et al^17^ reported more efficiency of 3D Accutomo CBCT (80 kVp, FOV=4 cm) system in the detection of the depth of proximal caries compared to the conventional method. In a study by Haiter-Neto, diagnostic sensitivity of CBCT technique was superior but not significant than the conventional method in the detection of dentinal occlusal caries. These differences might be attributed to different surfaces (proximal or occlusal) affected by caries in these studies and different CBCT systems with different fields of view used. Kalathinal showed better observer ability in CBCT technique for proximal caries depth detection. Therefore, it means that CBCT has better ability to determine the mineralization in dentinal structure, although deeper lesions are easily detected by other techniques too. Senel et al^18^ showed higher AZ value for CBCT (ILLUMA FOV 21.1- 14.2 cm) compared to the conventional and digital radiography in their study but no significant differences were found in the diagnostic accuracy. Zhang et al^19^ evaluated non-cavitated caries detection ability by two CBCT systems (ProMax 3D and Kodak 9000 3D) with digital (phosphor plates) and conventional images. The results showed that CBCT images were alittle more efficacious in detection of slight noncavitated caries,similar to digital and conventional radiographs and. In a study by Kayipmaz et al^20^ CBCT (Kodak 9500, FOV 9×15 cm) had better results in occlusal caries detection than the conventional and digital method but in the detection of proximal caries there was no significant priority. Some studies have shown the superiority of CBCT over other techniques in other fields in addition to caries detection.

The differences in the results of studies might be attributed to the use of various CBCT systems with different parameters, different FOVs (field of view), different kVps or different criteria for observer training. For example in this study a two-part criterion was used and in studies by Kalathinal and Zhang a five-part criterion was used. In this research Newtom 3G CBCT system was used but in Tsuchida's study 3D Accutomo system, and in Zhang research ProMax 3D and Kodak 9000 3D systems were used.

No doubt CBCT innovation provides a revolution in dentistry, which affords more and sufficient information in quality and quantity by only a little increase in patient dose, which is reasonable considering the data acqired. However, without any added information there is no reason for an increase in patient dose with the use of this technique. CBCT results in 3 to 7 times more patient dose than conventional method and it is time-consuming.

Considering its superiority only in the area under the ROC curve and similarity or deficiency in other factors, it is wise not to offer this CBCT system for proximal caries detection, especially by considering higher patient dose levels, which is more important in children during growth and development period.^21^ Farman^22^ emphasized ALARA as a principal basis in the diagnostic radiology and also in CBCT. This principle entails CBCT technique by reducing the basic projections or using new technologies in detector structures.^23^

It should also be reminded that the present study and all the studies mentioned above were in vitro studies carried out under ideal geometry. In addition, the teeth without any restoration were selected and it is obvious that presence of any metallic restorations in clinical situations might affect the quality of CBCT images. Therefore, further studies are recommended not only to clarify the accuracy of current systems but also to confirm and use future systems evaluated in vivo.^24^

## Conclusion

This study was undertaken to compare the diagnostic accuracy of cone beam computed tomography and conventional radiography in the detection of proximal caries. The results showed no advantage in CBCT systems. According to the results and in view of the higher patient dose, CBCT is not recommended for the detection of proximal caries.
